# Anoxic Brain Injury Presenting as Pseudosubarachnoid Hemorrhage in the Medical Intensive Care Unit

**DOI:** 10.1155/2017/9071482

**Published:** 2017-08-23

**Authors:** O'Dene Lewis, Samina Afreen, Supo Folaranmi, Marie Fidelia-Lambert, Vishal Poddar, Alicia Thomas

**Affiliations:** Howard University Hospital, 2041 Georgia Ave. NW, Washington, DC 20060, USA

## Abstract

Anoxic encephalopathy is frequently encountered in the medical intensive care unit (ICU). Cerebral edema as a result of anoxic brain injury can result in increased attenuation in the basal cisterns and subarachnoid spaces on computerized tomography (CT) scans of the head. These findings can mimic those seen in acute subarachnoid hemorrhage (SAH) and are referred to as pseudosubarachnoid hemorrhage (pseudo-SAH). Pseudo-SAH is a diagnosis critical care physicians should be aware of as they treat and evaluate their patients with presumed SAH, which is a medical emergency. This lack of awareness could have important clinical implications on outcomes and impact management decisions if patients with anoxic brain injury are inappropriately treated for SAH. We describe three patients who presented to the hospital with anoxic brain injury. Subsequent CT head suggested SAH, which was subsequently proven to be pseudo-SAH.

## 1. Introduction

Subarachnoid hemorrhage (SAH) is often a devastating clinical event with high mortality. An accurate diagnosis in patients with neurological impairment is essential [1]. In patients suspected of having SAH, nonenhanced computed tomography of the brain is the diagnostic imaging of choice. It has a high sensitivity for the detection of SAH, up to 98% [2] in patients scanned within 24 hours of symptom onset. CT head finding of high attenuation areas (HDAs) along the basal cisterns or cortical sulci is thought to be specific for SAH [3]. A similar appearance in the CT of the head may occur in the absence of blood in the subarachnoid spaces. This distinct finding is referred to as pseudosubarachnoid hemorrhage (pseudo-SAH).

Mimics of SAH include diffuse cerebral edema and purulent meningitis [3]. Spiegel et al. first described these findings in 1986 [4]. They reported that 10 patients with marked brain edema associated with a brain tumor or cerebral infarction showed SAH-like HDAs along the interhemispheric fissure and tentorium cerebelli on CT head. Autopsy done on all 10 patients showed no intracranial hemorrhage. In 1998, on reviewing head CT examinations of 100 comatose patients with brain edema, Avrahami et al. [5] found SAH-like findings along the cisterns and sulci in all of them and concluded that a CT diagnosis of SAH was unlikely. They proposed the term “pseudosubarachnoid hemorrhage” (pseudo-SAH) for this phenomenon.

In this report, we describe three patients with pseudo-SAH associated with diffuse cerebral edema and reviewed the literature regarding this imaging finding.

## 2. Patient 1

**Figure 1:**
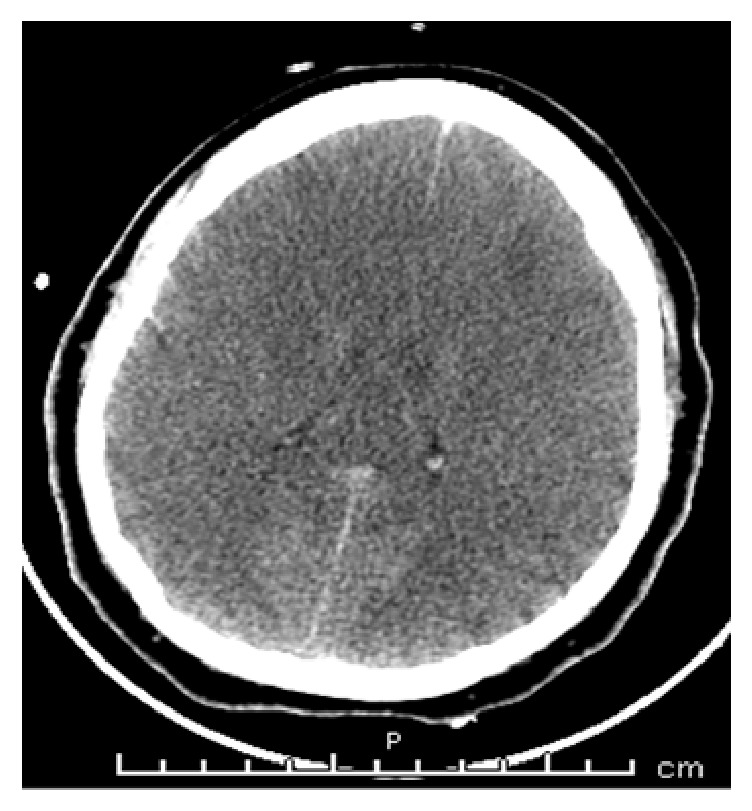
Noncontrast head computed tomography (CT) showing diffuse sulcal effacement and obliterated basal cisterns.

A 25-year-old African American female with an unknown past medical history was found unresponsive in her apartment. The patient was found to be severely hypoglycemic and was intubated for airway protection. She was admitted to the intensive care unit with a primary diagnosis of acute encephalopathy due to metabolic and/or septic etiology. On examination, she had a Glasgow Coma Scale (GCS) of 6T/15 with preserved brainstem reflexes and no lateralizing signs. An initial CT scan of her head appeared to be normal. A repeat CT scan of her head was performed in response to minimal improvement in the patient's neurological status. This revealed diffuse sulcal effacement and obliterated basal cisterns suggestive of SAH (Figure 1). Lumbar puncture showed an opening pressure of 29 cm and closing pressure of 25 cm. Cerebrospinal fluid (CSF) revealed the following: leucocytes 13/mm^3^ with a lymphocytic predominance, erythrocytes 10/mm^3^ without xanthochromia, normal protein and glucose, and negative Gram stain. Cerebral angiography was not performed. Lumbar puncture results were inconsistent with a diagnosis of SAH; thus, a diagnosis of SAH was rejected. Over the course of the hospital stay, there was a slight improvement in the patient's mental status to GCS of 8T/15. She subsequently had tracheostomy and percutaneous endoscopic gastrostomy (PEG) tube placement and was transferred to a long-term acute care hospital.

## 3. Patient 2

**Figure 2:**
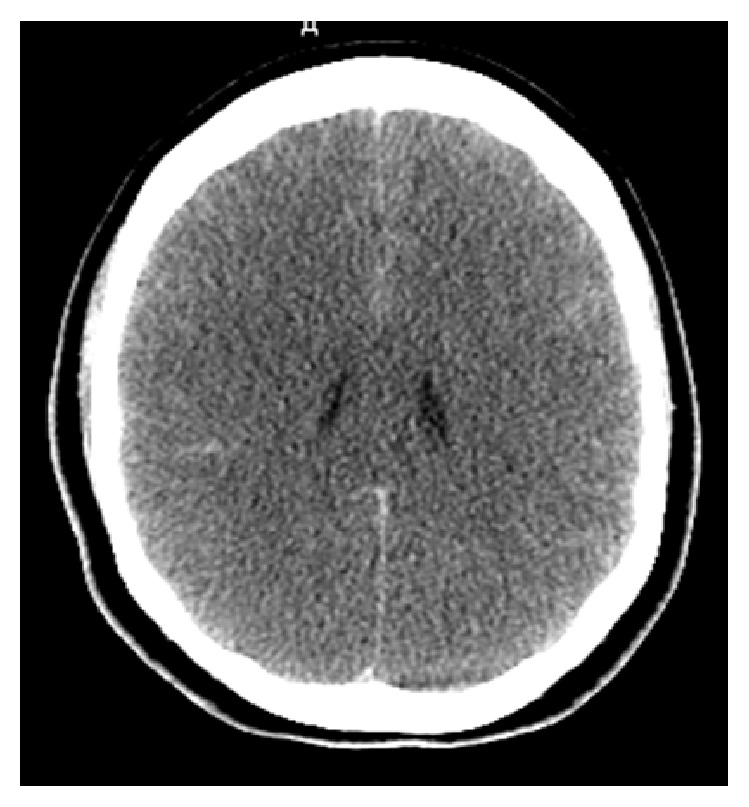
Noncontrast head computed tomography (CT) showing diffuse sulcal effacement, obliterated basal cisterns, and hyperdensity in the interhemispheric fissure.

 A 62-year-old African American female with a past medical history of tobacco abuse (no home medication) was brought in by an ambulance after a cardiac arrest. Her husband, a witness, reported generalized tonic-clonic seizure lasting approximately 5 minutes and she subsequently became unresponsive. The Emergency Medical Services (EMS) were called and cardiopulmonary resuscitation (CPR) was initiated. The patient had a return of spontaneous circulation after approximately 10 minutes and was intubated in the emergency vehicle and brought to the emergency room. On examination, the patient had a GCS of 3T/15. CT scan of the head showed diffuse sulcal effacement, obliterated basal cisterns, and hyperdensity in the interhemispheric fissure suggestive of SAH (Figure 2). The patient subsequently demised. Autopsy done revealed that the cause of death was a large inferior wall myocardial infarction with no evidence of SAH.

## 4. Patient 3

**Figure 3:**
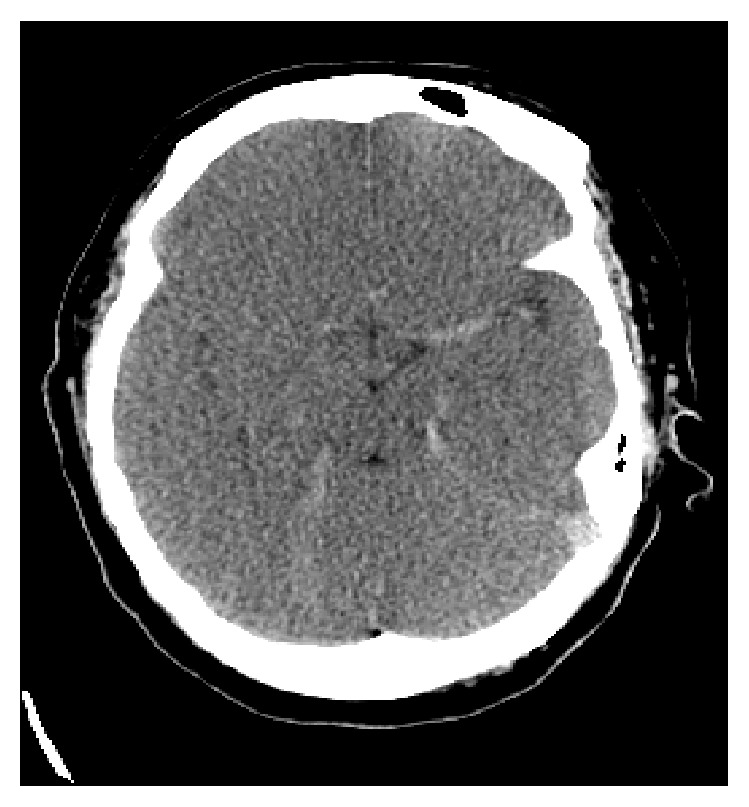
Noncontrast head computed tomography (CT) showing diffuse cerebral edema, with hyperattenuated cisterns and cerebral sulci.

 A 20-year-old African American female with a complaint of progressive shortness of breath 1 week earlier was brought in by an ambulance to the hospital after a cardiac arrest after she collapsed and lost consciousness in her room. Her roommate, who immediately called the EMS, witnessed the syncope. CPR was initiated, which continued en route to the hospital. After approximately 15 minutes of advance cardiac life support (ACLS), there was a return of spontaneous circulation. The patient was admitted to the ICU for post-cardiac arrest care. Based on the brief history, there was a high clinical index of suspicion for massive pulmonary embolism. An echocardiogram showed a dilated right ventricle suggestive of increased pulmonary artery pressure. CT angiography of the chest was not done due to hemodynamic instability and renal impairment. Based on the evidence highly suggestive of a massive pulmonary embolism, tissue plasminogen activator (TPA) infusion was started. She remained in a comatose state with no neurological response. CT scan of her head was performed which showed diffuse cerebral edema, with hyperattenuated cisterns and cerebral sulci, compatible with diffuse SAH (Figure 3). The patient at that time was too unstable to perform lumbar puncture or cerebral angiography. The TPA was stopped due to findings suggestive of SAH. A neurosurgery consult was requested, and the consultant reviewed the CT scan of the head with the radiologist. The attenuation coefficient at the basal cistern was 25–40 HU (Hounsfield unit) with a mean of 30–35 HU consistent with pseudo-SAH. The patient subsequently demised, thought to be due to a massive pulmonary embolism.

## 5. Discussion

A pseudo-SAH is a brain CT finding that is seen as high attenuation areas (HDAs) along the basal cisterns, the sylvian fissure, the tentorium cerebelli, or the cortical sulci in patients with severe brain edema, where no SAH is seen at autopsy or lumbar puncture [6].

To explain the origin of pseudo-SAH in association with cerebral edema, we considered the proposed causes of pseudo-SAH in other diseases. In purulent meningitis, bacterial toxins lead to the breakdown of the brain-blood barrier (BBB). The resultant BBB disruption allows proteinaceous material to leak into the subarachnoid space. However, CSF protein concentrations are sufficiently elevated to cause appreciable changes in CSF attenuation only in the most severe cases of meningitis [7–9]. The proteinaceous material in the subarachnoid space leads to vasogenic and cerebral edema, which contribute to the appearance of pseudo-SAH. Clinical history, CSF analysis, and enhancement of the basal cistern in postcontrast CT head can help differentiate between SAH and pseudo-SAH in this case [3].

Cerebral edema can lead to the cerebral cortex being displaced into the cerebrospinal fluid space and veins become congested due to the brain edema compressing the dural sinuses. The resultant subarachnoid spaces become relatively devoid of the hypoattenuated CSF and fill with a larger fraction of meninges and blood vessels than in the normal state, potentially increasing their CT attenuation [10]. With the development of cerebral edema, the attenuation of the brain parenchyma decreases concurrently [11, 12]. The decreased attenuation in brain parenchyma contributes to the pseudo-SAH appearance by increasing the conspicuity of the distended vasculature within the basal cisterns. Hypoxic ischemic encephalopathy after resuscitation from cardiopulmonary arrest, toxic encephalopathy due to valproate poisoning or bee sting, cerebral infarction, and/or severe head trauma can lead to cerebral edema and hence to the imaging finding of pseudo-SAH. In cases of cerebral edema, pseudo-SAH can be differentiated from SAH based on the attenuation coefficient in the basal cisterns. Attenuation coefficient is 21 to 44 Hounsfield units (HU) in case of pseudo-SAH, while it is 60–70 HU in cases with SAH. This difference was used to identify pseudo-SAH in our third patient in the absence of a lumbar puncture or autopsy findings. Following cardiopulmonary resuscitation, pseudo-SAH may develop within 3 days in approximately 20% of patients [6]. This indicates severe brain damage and suggests a poor prognosis, providing important information for deciding treatment strategies [6].

Agha and Al-Hakami described a young man with chronic renal failure who suddenly collapsed after starting hemodialysis. CT scan of the head showed severe brain edema and loss of sulci with hyperattenuated cisterns, suggestive of SAH. However, a four-vessel angiography did not show SAH. Further testing confirmed that the patient was brain-dead [13]. Given et al. described seven patients with diffuse cerebral edema and the appearance of SAH in the basal cisterns [10]. True SAH was excluded by lumbar puncture in four of the cases and by autopsy in three of the cases. Of the seven cases, only one patient survived. Al-Yamany et al. reported a case of a young man who suddenly collapsed after a drug overdose. CT scan showed an appearance of diffuse SAH, but autopsy performed 40 hours after his seizure revealed anoxic encephalopathy with no evidence of SAH [5]. Like these previously reported patients, our three cases exhibited clinical and radiological evidence of cerebral edema and increased intracranial pressure. In all three cases, the cisterns were effaced and the gray-white matter differentiation was obscured, compatible with diffuse cerebral edema. In the first case, CSF was not purulent and showed no evidence of hemorrhage. In the second case, autopsy did not show SAH similar to Al-Yamany et al. There was no definitive diagnostic test done on the third case. However, the imaging met criteria for pseudo-SAH based on the HDAs. Similar to the previously reported series, all of our patients had poor outcomes including 2 deaths and 1 with permanent neurologic sequelae.

Pseudosubarachnoid hemorrhage is considered a rare finding. It is important to recognize pseudo-SAH as it can lead to other diagnostic tests being performed, including angiographic studies to rule out vascular lesions and influence therapeutic decision-making in critically ill patients. Pseudo-SAH should be considered in patients with suspected SAH on CT scan of the head whose lumbar puncture does not demonstrate xanthochromia or in whom the clinical history is atypical for SAH but suggestive of another diagnosis, such as infection. However, due to the high morbidity and mortality from undiagnosed aneurysmal SAH, angiography should always be performed, unless a clear alternative diagnosis can be made.
